# Hardwiring antimicrobial resistance mitigation into global policy

**DOI:** 10.1093/jacamr/dlac083

**Published:** 2022-08-02

**Authors:** Kelly Thornber, Claas Kirchhelle

**Affiliations:** Department of Biosciences, University of Exeter, Stocker Road, Exeter EX4 4QD, UK; School of History, University College Dublin, Dublin 4, Ireland

## Abstract

In the wake of COVID-19, antimicrobial resistance (AMR) has become termed the ‘silent pandemic’, with a growing number of editorials warning that international momentum for AMR mitigation is being lost amidst the global turmoil of COVID-19, economic crises and the climate emergency. Yet, is it sufficient to now simply turn the volume of the pre-existing AMR policy discourse back up? Although existing AMR initiatives have previously achieved high levels of international attention, their impact remains limited. We believe it is time to critically reflect on the achievements of the past 7 years and adapt our AMR policies based on the substantial literature and evidence base that exists on the socioecological drivers of AMR. We argue that developing a more sustainable and impactful response requires a shift away from framing AMR as a unique threat in competition with other global challenges. Instead, we need to move towards an approach that emphasizes AMR as inherently interlinked and consciously hardwires upstream interventions into broader global developmental agendas.

Since the end of World War II, antimicrobial resistance (AMR) has received numerous waves of political attention.^1^ Much ink has been spilled on this topic: a quick web of science search of the terms antimicrobial resistance and antibiotic resistance revealed over 337 733 results (as of 15 April 2022), and between 1945 and 2020 there were at least 248 international reports published.^2^ The most recent wave of international attention for AMR culminated with the signature of the 2015 WHO Global AMR Action Plan^1^ (GAP) and released considerable resources—including the creation of ca. 102 AMR National Action Plans.^3^ Yet, 7 years after the Tripartite Agreement, AMR rates continue to increase,^4^ progress on infection prevention and control (IPC) programmes remains limited,^5^ and there has been little or no reduction in antibiotic consumption outside of a few high-income countries (HICs).^4,6–8^ Recent reviews highlight that the majority of resulting national AMR strategies are underfinanced and prioritize short-term reactive and surveillance/monitoring approaches rather than the longer-term preventive measures also recommended by the GAP.^9,10^ This disconnect is particularly pronounced in lower-income settings where AMR-specific interventions such as antibiotic usage monitoring and reduction targets achieve little in the face of other—more pressing—One Health and socioeconomic challenges, and many people still struggle to access antibiotics.^6,11–16^ Such mixed success on AMR policy implementation complicates recent calls^17^ for more action on AMR: should we now continue to follow down the policy tracks of 2015 or is it time to change our approach?

Fortunately, the last 7 years have provided us with an improved understanding of the biological and societal dimensions of the AMR challenge. In the social sciences there is now widespread acceptance that antimicrobials fulfil an essential infrastructural role within modern healthcare and food production systems. Structural dependencies on antibiotics mean that behavioural interventions appealing to the ‘rationality’ of individuals have limited effectiveness.^14^ Long-term AMR strategies must therefore move towards targeting the upstream environmental and socioeconomic factors shaping antibiotic dependency and broader disease ecologies.^18^ Weaning ourselves off antimicrobial ‘quick fix’ solutions^19^ includes improving, maintaining and expanding IPC programmes; investing in water, sanitation and hygiene systems (WASH); and broadening access to affordable healthcare. In food production, it also entails developing fair and equitable global commodity markets, reducing animal protein consumption and supporting sustainable food production.^20,21^ At the environmental level, ongoing advances in microbiology and One Health surveillance systems highlight the need to address antibiotic exposure and resistance selection in terrestrial and aquatic beyond farms and medical settings.^22^

Achieving these upstream AMR-sensitive interventions requires shifts in the way the AMR community frames policy challenges. Instead of emphasizing the distinctiveness of AMR or presenting resistance as a challenge of individual pathogens, particular settings, or communities, more efforts need to be directed at permanently embedding or hardwiring it into national and international sustainability and developmental agendas. At the international level, recent high-level reports^20,21^ have already begun this process by calling for the integration of AMR into the United Nations Sustainable Development Goals (SDG) and establishing a new SDG indicator (3.d.2), where the ‘Percentage of bloodstream infections due to selected antimicrobial-resistant organisms’ is used as an indicator of a country’s capacity to deal with human health risks.^20,21,23^ The joint framing of AMR as on par with other key planetary and developmental challenges is to be welcomed. However, more can be learnt from the climate community when it comes to communicating how the success of every SDG—ranging from poverty reduction to access to safe water—both influences and is dependent upon managing the threat that AMR poses to lives and livelihoods.

Resource-pooling needs to accompany this process of policy hardwiring. As we have learnt from the 2015 GAP, new AMR indicators and frameworks remain powerless if they are not supported by robust and equitable finance schemes. The moral burden of creating these schemes falls on HICs. Similar to HICs’ ethical duty to make greater investments to offset historic carbon emissions,^24,25^ we should acknowledge that low- and middle-income countries (LMICs) do not have access to the same levels of antibiotic effectiveness that HICs relied on when they created—and subsequently exported—the modern health and food production systems that shaped international development frameworks. Richer countries have an obligation to assist and collaborate with poorer neighbours when it comes to strengthening local health and agricultural infrastructures while simultaneously reducing systems’ vulnerabilities to AMR. HICs also need to pay greater attention to the role of global supply chains in driving antibiotic-intensive consumption practices and displacing problems of overuse and AMR pollution to poorer countries, for example in the demand for low-cost animal protein.^22,26–28^ In the case of LMICs, greater effort needs to be made to convince leaders that AMR mitigation is an essential part of future-proofing societies—as well as accepting that solutions will inevitably vary from those in HICs.^11,29^

Throughout its existence, the AMR challenge has competed for attention and resources with other socioecological systems challenges. This has often led to narrow and overly specific antibiotic policy interventions. Rather than fearing dilution and overemphasizing AMR’s distinctiveness, the AMR community should embrace the systems dimension of AMR and focus on hardwiring it into broader developmental agendas. This contextual approach will not only unlock synergies across SDGs, but also open the door for a more upstream mode of engagement that openly addresses both the complex ecological dimensions of AMR and the unequal burdens of AMR mitigation.

## References

[1] WHO . Global Action Plan on Antimicrobial Resistance. 2015. http://apps.who.int/iris/bitstream/10665/193736/1/9789241509763_eng.pdf? ua=1.

[2] Overton K , Fortané N, Broom A et al Waves of attention: patterns and themes of international antimicrobial resistance reports, 1945–2020. BMJ Glob Heal 2021; 6: e006909. 10.1136/bmjgh-2021-006909 PMC8573652 34740914

[3] WHO . Library of AMR National Action Plans. https://www.who.int/teams/surveillance-prevention-control-AMR/national-action-plan-monitoring-evaluation/library-of-national-action-plans.

[4] Murray CJ , Ikuta KS, Sharara F et al Global burden of bacterial antimicrobial resistance in 2019: a systematic analysis. Lancet 2022; 399: 629–55. 10.1016/S0140-6736(21)02724-0 35065702 PMC8841637

[5] WHO . Global Report on Infection Prevention and Control. 2022. https://www.who.int/publications/i/item/9789240051164.

[6] Browne AJ , Chipeta MG, Haines-Woodhouse G et al Global antibiotic consumption and usage in humans, 2000–18: a spatial modelling study. Lancet Planet Health 2021; 5: e893–904. 10.1016/S2542-5196(21)00280-1 34774223 PMC8654683

[7] Sriram A , Erta K, Kapoor G et al The State of the World’s Antibiotics in 2021. 2021. https://cddep.org/publications/the-state-of-the-worlds-antibiotic-in-2021/.

[8] WHO, Food and Agriculture Organization of the United Nations (FAO), World Organisation for Animal Health (OIE). Monitoring Global Progress on Antimicrobial Resistance: Tripartite AMR Country Self-Assessment Survey (TrACSS) 2019–2020: Global Analysis Report. 2021. https://www.who.int/publications/i/item/monitoring-global-progress-on-antimicrobial-resistance-tripartite-amr-country-self-assessment-survey-(tracss)-2019-2020.

[9] Tejpar S , Rogers Van Katwyk S, Wilson L et al Taking stock of global commitments on antimicrobial resistance. BMJ Glob Heal 2022; 7: e008159. 10.1136/bmjgh-2021-008159 PMC9121412 35589150

[10] Mcdonnell A , Klemperer K, Pincombe M et al Leveraging Purchasing Systems to Ensure Access, Stewardship, and Innovation: A Landscape Review of Current and Potential Market Structures for Antimicrobials. Center for Global Development. 2022. https://www.cgdev.org/publication/leveraging-purchasing-systems-ensure-access-stewardship-and-innovation-landscape-review.

[11] Charani E , Castro-Sanchéz E, Bradley S et al Implementation of antibiotic stewardship in different settings - results of an international survey. Antimicrob Resist Infect Control 2019; 8: 4–9. 10.1186/s13756-018-0460-8 30805181 PMC6373024

[12] Van Katwyk SR , Grimshaw JM, Nkangu M et al Government policy interventions to reduce human antimicrobial use: a systematic review and evidence map. PLoS Med 2019; 16: e1002819. 10.1371/journal.pmed.1002819 PMC6559631 31185011

[13] Munkholm L , Rubin O. The global governance of antimicrobial resistance: a cross-country study of alignment between the global action plan and national action plans. Global Health 2020; 16: 109. 10.1186/s12992-020-00639-3 33176810 PMC7656753

[14] Kirchhelle C , Atkinson P, Broom A et al Setting the standard: multidisciplinary hallmarks for structural, equitable and tracked antibiotic policy. BMJ Glob Health 2020; 5: e003091. 10.1136/bmjgh-2020-003091 PMC7513567 32967980

[15] Butcher A , Cañada JA, Sariola S. How to make noncoherent problems more productive: towards an AMR management plan for low resource livestock sectors. Humanit Soc Sci Commun 2021; 8: 287. 10.1057/s41599-021-00965-w

[16] Nayiga S , Willis LD, Staedk SG et al Reconciling imperatives: clinical guidelines, antibiotic prescribing and the enactment of good care in lower-level health facilities in Tororo, Uganda. Glob Public Health 2022; 27: 1–12. 10.1080/17441692.2022.2045619 PMC10083044 35220900

[17] Mendelson M , Sharland M, Mpundu M. Antibiotic resistance: calling time on the ‘silent pandemic’. JAC Antimicrobial Resist 2022; 4: dlac016. 10.1093/jacamr/dlac016 PMC8931486 35310572

[18] Buckee C , Noor A, Sattenspiel L. Thinking clearly about social aspects of infectious disease transmission. Nature 2021; 595: 205–13. 10.1038/s41586-021-03694-x 34194045

[19] Willis L D , Chandler C. Quick fix for care, productivity, hygiene and inequality: reframing the entrenched problem of antibiotic overuse. BMJ Glob Health 2019; 4: e001590. 10.1136/bmjgh-2019-001590 PMC6703303 31497315

[20] World Bank Group . Pulling Together to Beat Superbugs. 2019. https://documents1.worldbank.org/curated/en/430051570735014540/pdf/Pulling-Together-to-Beat-Superbugs-Knowledge-and-Implementation-Gaps-in-Addressing-Antimicrobial-Resistance.pdf.

[21] UN Interagency Coordination Group on Antimicrobial Resistance . No Time to Wait: Securing the Future from Drug-Resistant Infections. 2019. http://www.who.int/antimicrobial-resistance/interagency-coordination-group/final-report/en/.

[22] Wellcome, US CDC, UK Science & Innovation Network . Initiatives for Addressing Antimicrobial Resistance in the Environment. 2018. https://wellcome.org/sites/default/files/antimicrobial-resistance-environment-report.pdf.

[23] Sustainable Development Goals . Indicator 3.d.2: Percentage of Bloodstream Infections Due to Selected Antimicrobial-Resistant Organisms. https://sdg-indikatoren.de/en/3-d-2/.

[24] Botzen WJW , Gowdy JM, van den Bergh JCJM. Cumulative CO_2_ emissions: shifting international responsibilities for climate debt. Climate Policy 2008; 8: 569–76. 10.3763/cpol.2008.0539

[25] Baer P . Equity, greenhouse gas emissions, and global common resources. In: Climate Change Policy: A Survey. Stanford University, 2002; 393–408. http://stephenschneider.stanford.edu/Publications/PDF_Papers/15-Ch15%28393-408%29.pdf.

[26] Hogerzeil H , Frank G, Dinar-Farjon D et al Antimicrobial Resistance Benchmark Report 2021. 2021. https://accesstomedicinefoundation.org/media/uploads/downloads/61ee758c8c1e3_Antimicrobial Resistance Benchmark report 2021.pdf.

[27] Stenuick J-Y . Recommendations for Greener Human Medicines. 2022. https://noharm-europe.org/documents/position-paper-recommendations-greener-human-medicines.

[28] FAO . The FAO Action Plan on Antimicrobial Resistance 2021-2025. 2021. https://www.fao.org/documents/card/en/c/cb5545en/.

[29] Cañada JA , Sariola S, Butcher A. In critique of anthropocentrism: a more-than-human ethical framework for antimicrobial resistance. Med Humanit 2022. 10.1136/medhum-2021-012309 PMC9691817 35321873

